# Impact of invasive *Lantana camara* on maize and cassava growth in East Usambara, Tanzania

**DOI:** 10.1002/pei3.10090

**Published:** 2022-09-24

**Authors:** Amina A. Hamad, Japhet J. Kashaigili, Sandra Eckert, René Eschen, Urs Schaffner, John Richard Mbwambo

**Affiliations:** ^1^ School of Forestry, Wildlife and Tourism Sokoine University of Agriculture Morogoro Tanzania; ^2^ Center for Development and Environment University of Bern Bern Switzerland; ^3^ Institute of Geography University of Bern Bern Switzerland; ^4^ CABI Delémont Switzerland; ^5^ Lushoto Silviculture Research Center Tanzania Forestry Research Institute Lushoto Tanzania

**Keywords:** allelopathy, crop growth, East Usambara, invasive alien plant species, *Lantana camara*, soil nutrients and microorganisms

## Abstract

The impacts of invasive alien plant species on native plants are generally well documented, but little is known about the mechanisms underlying their impacts on crop growth. A better understanding of immediate as well as legacy effects and of direct and indirect impacts of invasive alien plant species is essential for an improved management of invaded cropland. We investigated how *Lantana camara* impacts the growth of two subsistence crops (maize and cassava) through competition for resources, allelopathy and the indirect plant–plant interactions. We carried out two pot experiments using soils from invaded abandoned, invaded cultivated and non‐invaded cultivated crop fields. In the first experiment maize and cassava were grown alone or together with *L. camara* and half of the pots were treated with activated carbon to suppress allelochemicals. The effect of the soil microbial community on *L. camara*—crop interactions was assessed in a second experiment using autoclaved soil with 5% of soil from the three soil types. We found that *L. camara* reduced the growth of maize by 29%, but cassava was not affected. We did not find evidence of allelopathic effects of *L. camara*. Inoculation of autoclaved soil with microorganisms from all soil types increased biomass of cassava and reduced the growth of maize. Because *L. camara* only caused impacts when growing simultaneously with maize, the results suggest that removal of *L. camara* will immediately mitigate its negative impacts on maize.

## Graphical abstract

The impacts of invasive alien plant species on native plants are generally well documented, but little is known about the mechanisms underlying their impacts on crop growth. We investigated how Lantana camara impacts the growth of maize and cassava through competition for resources, allelopathy and the indirect plant‐plant interactions. Because L. camara only caused impacts when growing simultaneously with maize, the results suggest that removal of L. camara will immediately mitigate its negative impacts on maize.

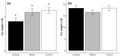

## 1 INTRODUCTION

Over the last decades, an increasing number of studies have documented the diverse and vast scale of the negative impacts of invasive alien plant species (IAPs) on ecosystem services (Charles & Dukes, 2007; Ipsita et al., 2014; Pratt et al., 2017; Vilà et al., 2011). Alien plant species become invasive when they spread from their original place of introduction to other areas and cause economic or environmental damage, for example in crop or livestock production, or through reductions in biodiversity or access to water resources. IAPs are known to establish particularly well in disturbed habitats. Farming practices such as shifting cultivation (a form of disturbance) may thus facilitate alien plant invasions (Petit et al., 2011). Even though impacts of IAPs on crop production have been well documented, the mechanisms through which these species affect crop growth are not well understood (Ahmed et al., 2007; Skurski et al., 2014). Yet, it is important to understand the mechanisms of impact of IAPs on crop growth for the development of targeted crop management.

The effects of IAPs on crops may be caused by a variety of mechanisms, which can act immediately while the plants co‐occur, or legacy effects may occur after the invader is removed (Grove et al., 2012). Equally, effects can be due to direct interactions, or they can be mediated through changes in biotic or abiotic soil properties induced by the IAPs growing in the soil (indirect effects; van der Putten et al., 2013). IAPs may immediately interact with crops and reduce their yield as a result of competition for resources, such as nutrients or light, that would otherwise be available for the crop (Gallandt & Weiner, 2015). For example, rapid growth of IAPs causes shading which can reduce the fitness of neighboring plants (Page et al., 2010). Allelopathy can inhibit growth of neighboring plants immediately through changing soil chemical properties as a result of the root exudates of IAPs, or via phytotoxic root exudates (Callaway & Aschehoug, 2000). Phytotoxic root exudates can mediate negative plant–plant interactions only if present at sufficient concentrations to affect plant growth and survival. The ecological relevance of phytotoxic root exudates also depends on the susceptibility of the plants with which the allelopathic plants coexist (Bais et al., 2006).

Allelochemicals bind to soil particles and some allelochemicals can persist in soil when the producing plant is no longer there, and may create a legacy effect (Del Fabbro & Prati, 2015). However, such persistent allelopathic effects have rarely been explored (Grove et al., 2012; Tian et al., 2007). Legacy effects of IAPs may also be mediated by changes in the abiotic or biotic soil environment, that is, via plant–soil feedback (Ehrenfeld, 2010). For example, invasive plants often increase the available nitrogen (N) and total N content of the soil (Vilà et al., 2011), which may improve the environment for individuals of the same IAPs and may increase their competitive interactions (e.g. Osunkoya & Perrett, 2011). Changes in the soil microbial community in response to the allelochemicals released by IAPs, can be either beneficial or detrimental for the growth of individuals of the same IAPs and other plant species growing in the soil. These changes in the soil properties can affect growth of other plants species even after the IAP has been removed or has died (Lankau et al., 2014). The effect of changes in the soil microbial community has been illustrated by (Wolfe et al., 2008), who found that the invasive *Alliaria petiolata* L. lowered growth of native tree species through phytotoxin‐induced reductions in arbuscular mycorrhizal fungal colonization of the tree roots.

*Lantana camara* L. (Verbenaceae) is native to tropical America and was introduced in different parts of the tropics, example, India and Eastern Africa. It became a popular hedge and garden plant in early 20th century due to its introduction in botanical gardens in different European colonies (Dawson et al., 2008). The species invades a wide range of habitats, but grows best in open, disturbed ecosystems, along forest edges, and roadsides (Sharma et al., 2005). *L. camara* has become widely established in the East Usambara Mountains of Tanzania, which are dominated by tropical montane forests interspersed with villages and agricultural areas. The farmers in the region associate *L. camara* with lower crop yield if it is present on cultivated land. These claims were also reported by Shackleton et al. (2017) who assessed the perception of pastoral and agro‐pastoral communities of Uganda and claimed 26%–50% reductions in crop yield as a result of *L. camara* presence. On the other hand, farmers also associate fields that were invaded with *L. camara* prior to cultivation with better crop yield, compared to non‐invaded fields (A.H., pers. obs.). Several studies have reported higher nitrogen content of soil with *L. camara* growing in it (Fan et al., 2010; Osunkoya & Perrett, 2011; Sharma & Raghubanshi, 2009), but to our knowledge there is no experimental evidence that links the higher nutrient content of *L. camara* invaded soil to improved crop growth. There are also studies claiming allelopathic effect of Lantana on crop plants. While most of these studies were done in artificial environments, some were done under natural conditions (Gentle & Duggin, 1997).

Mechanisms of impacts, particularly whether the impact occurs in the presence of the IAP or represents the legacy of the IAP, may affect management decisions with respect to control and prevention of IAPs impacts in agricultural systems. Therefore, the aim of this study was to understand the mechanisms of impacts of *L. camara* on the growth of maize (*Zea mays* L.) and cassava (*Manihot esculenta* Crantz), in East Usambara, Tanzania. Specifically, we tested in two parallel pot experiments whether the effect of *L. camara* on maize and cassava growth was mediated by (a) competition for limiting resources, (b) direct interference, or (c) indirectly through altered microbial interactions. We also assessed the effect of the native shrub *Whitfieldia elongata* (P. Beauv.) De Wild. &T. Durand (Acanthaceae), to test if the impact of *L. camara* is different from that of a native shrub that is common and widespread in the area.

## 2 METHODS

### 2.1 Study area

The East Usambara mountains in Tanzania comprise a network of 18 forest blocks covering an area of 263 km^2^ at an altitude range of 250–1506 m above sea level (Burgess et al., 2007). Among the 18 forest reserves, Amani Nature Reserve (ANR) is the largest (Hamilton & Bensted‐Smith, 1989), covering an area of 8380 ha and lies between 300 to 1128 m above sea level. The ANR forest ecosystem has a close interaction with people, as two of the 19 villages bordering the reserve are enclaved within the forest (Mpanda et al., 2011). The main economic activity of the inhabitants is cash‐ and food crop cultivation. The main cash crops include sugar cane and spices such as cardamom, cinnamon, cloves, and black pepper. The cultivated food crops include cassava, beans, sweet potatoes, and maize. While the two main annual crops cultivated locally are cassava and sugar cane, we selected maize and cassava for studies because the two are main food crops in Tanzania. In Amani, particularly maize fields are vulnerable to *L. camara* invasion as they are left fallow for some months after harvesting.

### 2.2 Plant material

Cassava stems from a single variety were obtained from a farmer in the study area in May 2017. The exact variety was unknown, as many farmers propagate cassava from stems collected from farms with previous good harvests without regard for the exact variety. For the experiment, cassava cuttings of approximately 30 mm diameter and 20 cm length were used. After consulting with the farmers, seeds of the main maize variety used in the study area were purchased (SEED.CO hybrid maize seed SC 719). Cuttings of the native shrub *W. elongata* and of *L. camara* were collected in the wild around the study area at the same time as the cassava cuttings. The use of *W. elongata* was suggested by a botanist, as it is a native shrub used as ornamental and widely distributed in Eastern Africa. It grows in open to moderately shaded areas and can be propagated through cuttings like *L. camara*. The cuttings of both shrubs had an average height of 30 cm and a diameter of 10 mm. The cuttings of *L. camara* and *W. elongata* were longer than that of cassava to simulate the field condition, as the shrubs outgrow crops in the field.

### 2.3 Soil material

The soil used in the pot experiments was collected from fields that differed in their history of *L. camara* invasion and agricultural management: (1) “Abandoned” soil was collected from fields that had been fallow for at least 3 years with a *L. camara* coverage >75%; (2) “Invaded” soil was collected from cultivated fields that had not been weeded during the previous 3 months and that had c. 40% *L. camara* cover; and (3) “Non‐invaded” soil was collected from cultivated fields that had not been weeded during the previous 3 months and contained no *L. camara* plants. Soil from each type was collected from six different farms. To avoid a bias caused by the previously grown crop type, we sampled each soil type soil from three locations previously cultivated with maize, the other three with cassava. Soil was collected by first removing the litter and then collecting loose soil from the top soil layer down to about 50 cm depth using a shovel. The soil from each location was then mixed and stored in woven plastic bags. Samples from each soil type and location were analyzed for pH (pH‐KCL), total nitrogen (Kjeldahl), organic carbon, carbon‐nitrogen ratio, potassium, phosphorous (Bray and Kurtz 1), calcium, magnesium, and sodium content according to standard laboratory protocols (Ministry of Agriculture and Fisheries, 2016). The soil samples to be autoclaved were obtained from a single location without *L. camara* to keep the nutrient content constant and transferred to the Mlingano laboratory, Tanga District, for autoclaving at 121°C for 30 min. Autoclaving was repeated once per day for 3 days. The soil samples were collected 7 days prior to setting up the experiment to give time for autoclaving.

### 2.4 Study 1—Effects of shrubs and allelochemicals

**FIGURE 1:**
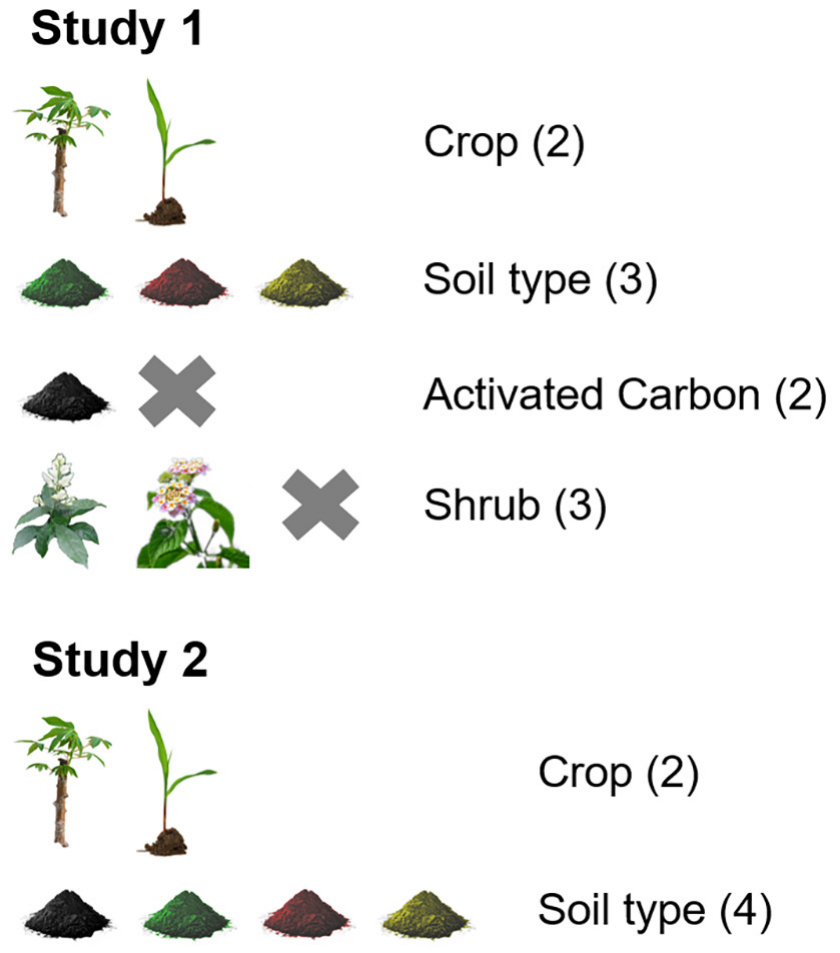
An overview of the experimental factors influencing the growth of maize and cassava in the two studies. Numbers in brackets indicate the number of levels for each factor and crosses indicate untreated control treatments. The study design was full factorial and there were six replicate origins per soil type, resulting in a total of 216 pots in study 1 and 48 pots in study 2.

To assess the effects of soil type, the presence of *L. camara* or *W. elongata* and allelochemicals on the growth of the two crops (maize and cassava), a full‐factorial experiment was set up in 2 L pots (Figure 1). The six locations where the soil samples were collected were used as replicates. One third of the pots were filled with soil from each soil type. Activated carbon (2% v/v; Charcoal activated Art. 2690; Loba Chemie) was added to half of the pots by mixing the required amount of activated carbon with the soil prior to filling the pots. Activated carbon can absorb organic compounds including allelochemicals and can reduce or even eliminate the negative effects of allelochemicals (Callaway & Aschehoug, 2000, but see Lau et al., 2008). The experiment comprised 216 pots. One cutting of *L. camara* each was then planted in one third of the pots and one cutting of *W. elongata* in another third of the pots, while the remaining pots were not planted with any shrub. The fresh weight of the cuttings was recorded prior to planting. Half of the pots, each containing either *L. camara* or *W. elongata* (*n* = 108), were then planted with three seeds of maize, the other half for each pot with one cutting of cassava. The fresh weight of cassava cuttings was measured before planting. After 1 month the germinated maize plants were reduced to one per pot. The pots were placed in a completely randomized order in an experimental garden at the Amani Botanical Garden, Tanga District, Tanzania (5°06′2.5′′S, 38°37′46′′E; altitude 924 m a.s.l.) in mid‐May 2017 and were watered daily. After 4 months the crops and shrubs were harvested. Shrub and crop roots were carefully disentangled using water to completely remove the soil and loosen the roots. Each crop and shrub from a pot was then stored in a separate paper bag and labeled. Aboveground and belowground fresh biomass were measured within 1 day from harvesting. Crops were then taken to the laboratory and oven dried at 80°C for 24 h. Then the total dry biomass of the crops was measured, because not many roots were formed and after drying the root weight was almost zero.

### 2.5 Study 2—Effects of microbial community

To assess the effects of soil microorganisms on the growth of maize and cassava four soil treatments were used: (a) 100% autoclaved soil, 95% autoclaved soil plus 5% of soil from either (b) abandoned, (c) invaded or (d) non‐invaded fields (Figure 1). Adding 5% soil that was not autoclaved was done to introduce microorganisms from the three soil sources, while other soil components such as nutrients remained largely unchanged (Shaw et al., 1999). The addition was done by mixing the autoclaved and untreated soil samples before filling the pots. The six fields from which each soil type was collected were used as replicates. In half of the pots for each treatment one cassava cutting was planted and in the remaining pots three maize seeds were sown as in Study 1, resulting in a total of 48 pots. The fresh weight of each cassava cutting was recorded prior to planting. The pots were placed in the experimental garden in completely randomized order during first week of June 2017 and watered daily thereafter. After a month maize plants were reduced to one per pot. After 4 months the crops were harvested and the same measurement procedure as in Study 1 was applied.

### 2.6 Statistical analysis

Soil chemical properties were analyzed using generalized linear models with the individual chemical parameters as response variables and the soil type as explanatory variable.

The effects of soil type, allelochemicals and the presence of shrubs on biomass of maize and cassava (Study 1) were tested using a generalized linear mixed effects model with soil type, activated carbon, crop type, and shrub treatments as fixed factors. The location where the soil was collected was included as random effect. Generalized linear models with dry biomass as response variable and crop species and soil chemical parameters as explanatory variables were used to test the effects of the soil chemistry on crop biomass. For this analysis, pots where AC was added and where *L. camara* or *W. elongata* was present were excluded. We calculated the Relative Efficiency Index (REI; Connolly, 1987) as a measure of the relative competitive ability of the crops; the higher REI the stronger (higher relative competitive ability). REI was calculated as (ln(Final crop weight)‐ln(Initial crop weight)) − (ln(Final shrub weight) − ln(Initial shrub weight)). The effects of shrub type on REI were analyzed using similar linear mixed effects models as above, but for the crops separately, as the data showed a bimodal distribution as a result of the differences in crop weight. The relationship between the increase in crop weight and the change in shrub weight was assessed using a Pearson correlation.

The effects of microbial community composition of the soil types on crop growth (Study 2) were analyzed using a generalized linear model with biomass as response variable, soil treatment and crop type as fixed factors, and locations where the soil was collected as random effect. Least square means was used to test linear contrast within treatments if significant effects were found among levels within a given treatment.

## 3 RESULTS

### 3.1 Soil characteristics

The carbon to nitrogen ratio (C:N) was higher in non‐invaded soil than in the soil samples collected from abandoned and invaded fields (*F*_2,40_ = 7.4, *p* = .006; Table 1). However, the C:N ratio in soil samples of abandoned and invaded fields was not different (*p* > .05). Similarly, the organic carbon content of soil samples of non‐invaded fields was higher than that in soil samples of invaded and abandoned fields (*F*_2,40_ = 7.5, *p* = .006). Total nitrogen content did not differ among the three soil types (*p* > .1), indicating that the difference in the C:N ratio was due to differences in carbon content. A positive correlation between the organic carbon content and C:N ratios of the soils was found (*t* = 3.98, *p* = .001; Figure S1). The other parameters (P, K, Ca, Na, Mg, pH and EC) did not differ significantly among the three soil types (all *p* > .1).

**TABLE 1:** Summary of the abiotic characteristics of the three soil types. Numbers indicate means of six soil origins ± SE

Soil chemistry	Soil type		
	Abandoned	Invaded	Non‐invaded
Total *N* (%)	0.05 ± 0.00	0.06 ± 0.00	0.05 ± 0.00
C_Org_ (%)	0.69 ± 0.05	0.81 ± 0.05	0.95 ± 0.03
C:N	14.23 ± 0.97	13.90 ± 0.91	18.54 ± 0.97
Na (mg/kg)	1.89 ± 0.28	4.36 ± 0.26	2.89 ± 0.55
K (mg/kg)	0.39 ± 0.06	0.39 ± 0.08	0.43 ± 0.10
P (mg/kg)	6.20 ± 0.38	5.22 ± 0.53	7.14 ± 0.77
Ca (mg/kg)	8.11 ± 0.43	6.09 ± 0.77	6.63 ± 0.74
Mg (mg/kg)	2.28 ± 0.23	1.56 ± 0.42	1.82 ± 0.10
pH_KCl_	5.41 ± 0.10	5.29 ± 0.12	5.30 ± 0.17

### 3.2 Study 1—Effect of shrubs, soil source and allelochemicals

**FIGURE 2:**
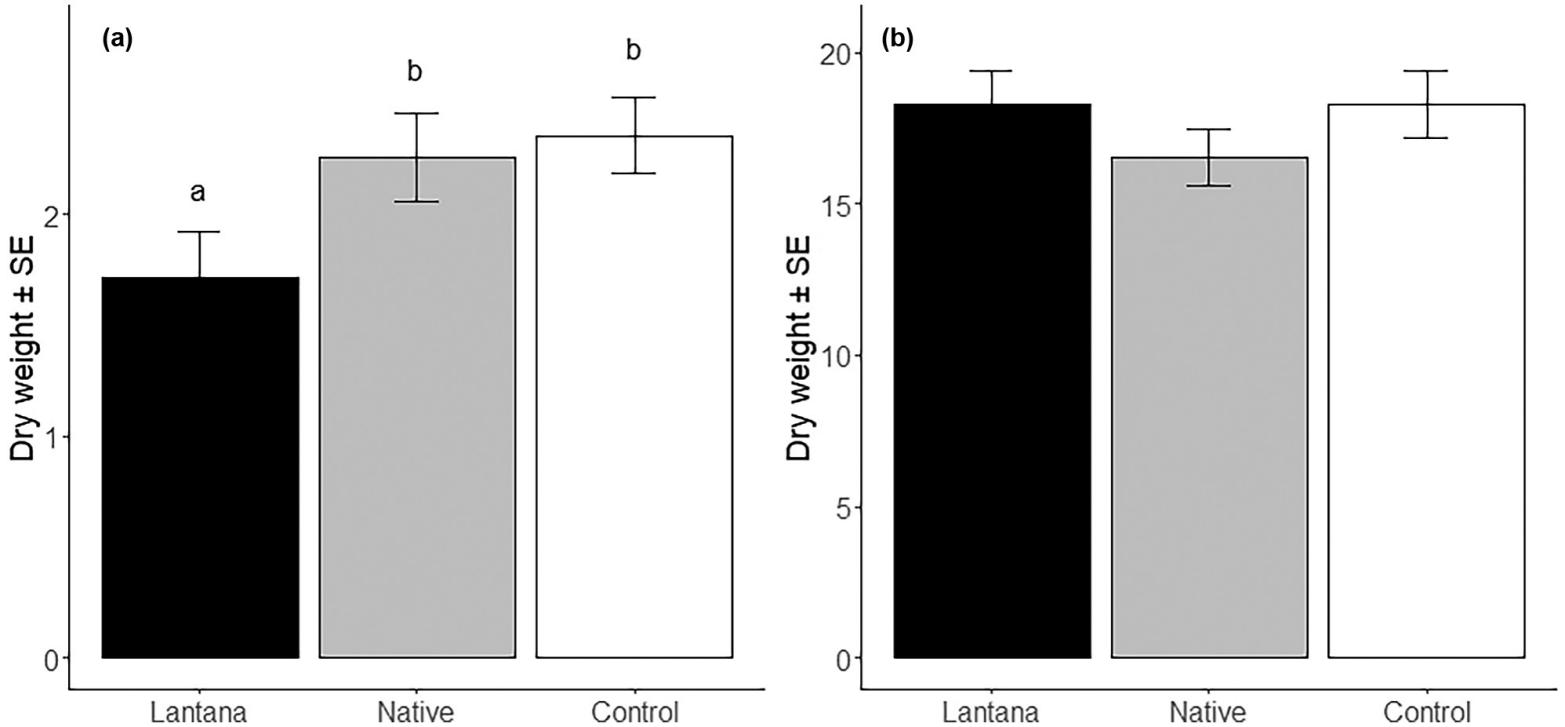
Dry weight of (a) maize and (b) cassava, grown in pots together with *Lantana camara*, *Whitfieldia elongata*, and no shrub (control). Error bars indicate one standard error and letters above bars indicate significant differences among means.

The dry weight of cassava was larger than that of maize (*p* < .001; Table 2; Figure 2). Furthermore, a significant interaction between the crop and shrub treatments was found (*p* = .048), indicating that the crops responded differently to the presence of the two shrubs (Figure 2). Hence, we analyzed the two crops separately and found that the difference between crop and shrub treatments was significant in maize, while it was not in cassava (*F*_2,70_ = 3.46, *p* = .037 and *F*_2,78_ = 0.56, *p* = .573, respectively; Figure 2): Presence of *L. camara* suppressed maize growth by 29% (*p* = .05) compared to when maize was growing alone or in the presence of *W. elongata* (Figure 1). Presence of *W. elongata* did not have a significant effect on maize biomass (*p* = .7). Additionally, we looked at the relationship between crop and shrub growth by comparing the change in shrub weight with crop biomass. No relationship between crop and shrub growth was found (Pearson correlation: *p* > .2) and in both crops no significant differences in REI between shrub species were found (*p* > .1; overall mean for maize and cassava 3.47 ± 0.18 and 0.37 ± 0.04, respectively). There was no difference in crop biomass among the soil types (*p* = .367; Table 1) and the addition of AC had no significant effect on biomass (*p* = .428). A significant negative relationship between the C:N of the soils and crop biomass was found (*F*_1,14_ = 4.84, *p* = .045), but no relationship with organic carbon content. Marginally significant positive relationships between belowground fresh crop biomass and Total N and the N:P ratios of the soils were found (*p* = .090, *r* = .20 and *p* = .077, *r* = .27; Figure S1), suggesting that N was the limiting nutrient for growth of the two crops. Analysis of the aboveground and belowground fresh weights revealed different treatment effects on belowground weight than on aboveground weight: a significant soil type by crop interaction was found for aboveground fresh weight (Table 2), which was due to lower maize shoot weight when grown in soil without Lantana than in the other two soils, whereas no such differences were seen in cassava. Belowground biomass was affected by soil type and shrubs; similar to total dry weight, root fresh weight was lowest when the plants were grown with Lantana and highest when no shrub was present (the root weight in presence of W. elongata was intermediate). Root fresh weight was lowest when plants were grown in control soil; the fresh root weight of plants grown in invaded or abandoned soil was not different. No effect of AC on aboveground or belowground weights, and no different effect on the two crops were found.

**TABLE 2:** Summary table of the effects of crop type (maize or cassava), activated carbon (AC), the presence of shrubs (none, *Lantana camara* or *Whitfieldia elongata*), soil type (non‐invaded vs. abandoned vs. invaded), and interactions between these on dry biomass. Shown are degrees of freedom (denominator, numerator), *F*‐ratios and *p*‐values (*p* < .05 is highlighted)

Factor	*df*	Total dry weight		Aboveground fresh weight		Belowground fresh weight	
		*F*	*p*	*F*	*p*	*F*	*p*
Crop	1161	617.17	**<.001**	1059.8	**<.001**	45.06	**<.001**
AC	1161	0.62	.432	950.3	**<.001**	2.44	.120
Shrub	2161	2.59	.078	0.9	0.335	1.00	.320
Soil type	2,15	0.42	.662	1.0	0.373	4.78	**.010**
Crop × AC	1161	0.02	.904	1.9	0.178	9.88	**.002**
Crop × shrub	2161	3.04	**.050**	0.0	0.999	0.25	.618
AC × shrub	2161	0.10	.902	1.4	0.256	0.64	.530
Crop × soil type	2161	0.31	.733	0.6	0.547	0.04	.959
AC × soil type	2161	0.13	.878	5.8	**.004**	0.76	.471
Shrub × soil type	4161	0.22	.928	0.8	0.449	1.77	.174
Crop × AC × shrub	2161	0.67	.512	0.4	0.836	0.94	.445
Crop × AC × soil type	2161	1.70	.186	1.1	0.348	1.58	.209
Crop × shrub × soil type	4161	0.67	.611	0.9	0.419	1.96	.144

### 3.3 Study 2—Effect of microorganisms

**FIGURE 3:**
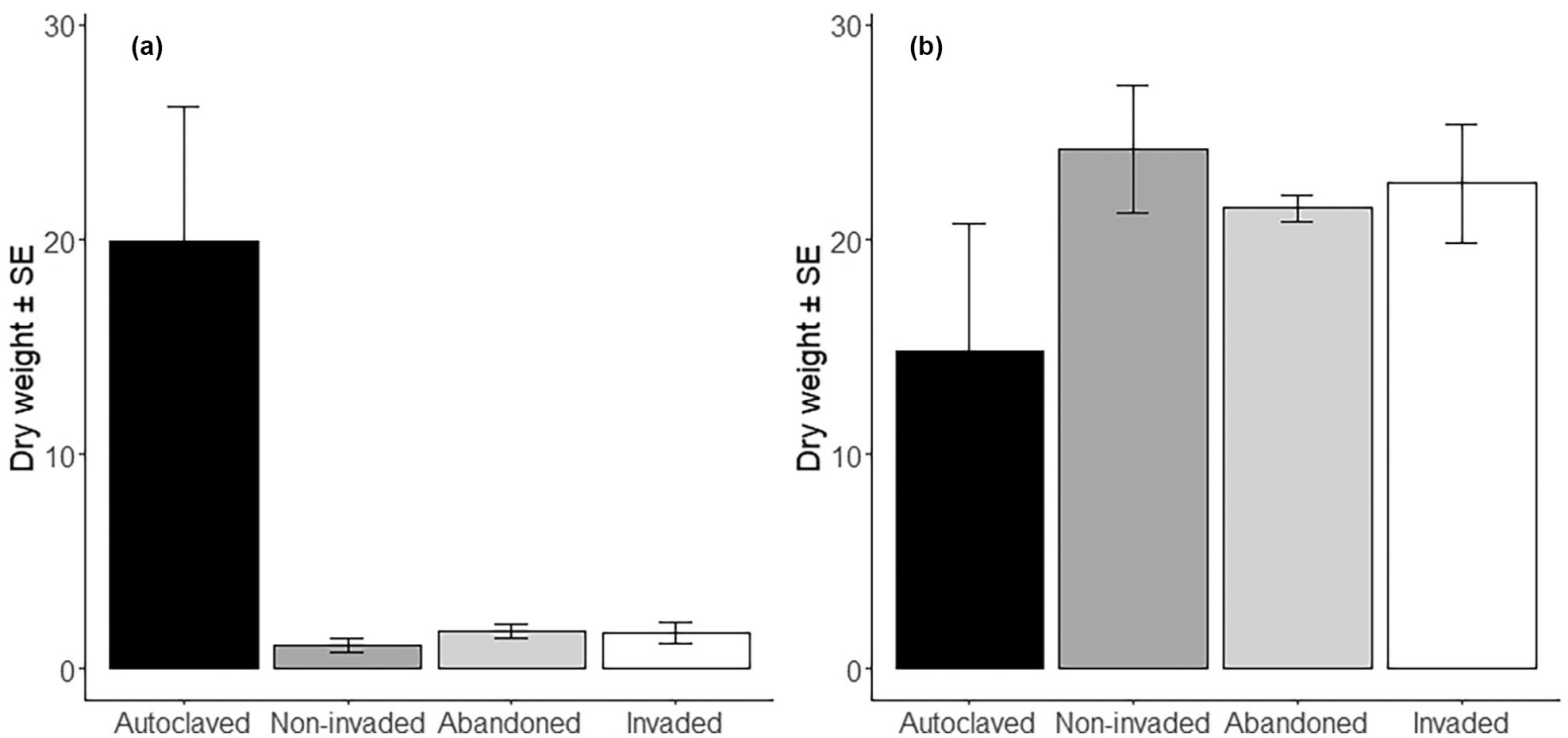
Dry weight of (a) maize and (b) cassava, grown in pots with autoclaved soil together with 5% (v/v) untreated soil from abandoned, invaded or non‐invaded fields. Autoclaved indicates pots to which no untreated soil was added. Error bars indicate one standard error.

Biomass of the two crops was affected differently by the soil treatments (*p* < .001; Table 3). Maize biomass decreased by 90% when microorganisms were introduced in autoclaved soil (*p* < .001). In contrast, cassava biomass was 35% higher when microorganisms were introduced in autoclaved soil, but the effect of microorganisms on cassava biomass was only marginally significant (*p* = .07; Table 2, Figure 3). When autoclaved soil was omitted from the analysis a non‐significant difference in maize and cassava biomass among the three soil types was found (*p* > .1), indicating that the soil microbial community in the three soil types had no different effect on the growth of the two crops. A significant interaction between crop and soil treatments on aboveground fresh weight was found (Table 3), which was the result of similar crop weight on autoclaved soil and while addition of microorganisms of any soil type reduced maize above ground weight, aboveground weight of cassava was not affected. No significant differences in root fresh weight were found between crops or soil treatments.

**TABLE 3:** The effects of crop, soil treatment and interactions between these on dry biomass. Shown are degrees of freedom (denominator, numerator), *F*‐ratios and *p*‐values (*p* < .05 is highlighted)

Factor	*df*	Total dry weight		Aboveground fresh weight		Belowground fresh weight	
		*F*	*p*	*F*	*p*	*F*	*p*
Crop	1,17	145	**<.001**	119	**<.001**	0.07	.791
Soil treatment	3,19	0	.992	0	.969	0.63	.606
Crop × soil type	3,17	19.9	**<.001**	16.5	**<.001**	0.79	.516

## 4 DISCUSSION

### 4.1 Immediate effects

In our experiment (Study 1), the growth of maize in the presence of *L. camara* was reduced by 29% compared to maize grown alone or in combination with *W. elongata*. To our knowledge, this is the first study to demonstrate and quantify the impact of *L. camara* on crop growth in semi‐natural conditions. This is important, because the economic impact of IAS is understudied, especially in Africa (Diagne et al., 2021; Eschen et al., 2021). We considered competition for limiting resources and allelopathy as potential mechanisms for this immediate *L. camara* effect, but found limited support for either. Root fresh weight was lower if plants were grown together with *L. camara*, which may be an indication of competition through depletion (Schenk, 2006), and the absence of an effect of AC suggests that it was not contest competition such as allelopathy, but the effect was not found in aboveground fresh biomass or total dry biomass, indicating that the overall effect on crop growth was minor. We found weak positive relationships between crop biomass and nitrogen and N:P ratio, suggesting that nitrogen was the limiting nutrient for growth of maize and cassava in these soils. Sun et al. (Sun et al., 2013) studied mechanisms explaining interactions between invasive *Centaurea stoebe* and competitors from the native and exotic ranges of the species and found a negative relationship between the biomass of *C. stoebe* and competitors from the native range. The authors concluded that competition for resources occurred in the pots where the species were grown together. Differences in REI between pots with competitors from the native or exotic range were explained by inherent differences in relative competitive ability of species that had a shared history with the invader or not. In our study, the absence of a relationship between crop and shrub growth between pots with *L. camara* or pots with *W. elongata* suggests that competition for soil nutrients did not cause the reduction in maize biomass and the absence of differences in REI points to a similar relative competitive ability of *L. camara* and the native shrub species.

If competition played a role, one would expect that growth of cassava would also have been affected and, it would also have occurred in the presence of the native *W. elongata*, which was not the case. Moreover, the results suggest that the growth of the shrubs (and perhaps cassava) was not affected by the nutrient content of the soil. The weight of the shrub cuttings decreased during the experiment, even though leaves and roots were produced by all cuttings. This weight reduction may have been due to investment of resources by the planted cuttings in the formation of roots and leaves, but we did not assess the biomass of new shoots or roots after planting separately. Despite the significant reduction of maize growth in the presence of *L. camara*, the results of our studies do not allow firm conclusions about the mechanisms that underlie the impact. Nutrient addition studies would be necessary to determine whether competition for nutrients occurred.

The presence of activated carbon in pots with *L. camara* growing together with either of the two crops had no significant effect on their biomass, which suggests that no immediate allelopathic effects occurred in our study. This appears in contrast to studies that found that *L. camara* has an allelopathic effect. Several studies showing an allelopathic effect on crop growth were conducted in Petri dishes or pots using *L. camara* leaf extracts (Ahmed et al., 2007; Ipsita et al., 2014; Mishra, 2014) or dry shoot residue (Mersie & Singh, 1987). Such studies have revealed that *L. camara* leaf extracts can have an inhibitory effect on shoot and root elongation, as well as biomass of various crops and weed species (Ahmed et al., 2007). *L. camara* dry shoot residues were found to strongly reduce growth of maize in these artificial conditions (Mersie & Singh, 1987). Experiments conducted under artificial conditions may exaggerate the allelopathic effects of the studied plants (Hierro & Callaway, 2003) and do not necessarily reflect natural conditions (Inderjit et al., 2005; Inderjit & Weston, 2000). In a field study by Gentle and Duggin (1997), germination and early biomass accumulation of two Australian native tree species was tested in soil where *L. camara* was either removed and activated carbon added, burnt, or cut and the cut branches left in place and the results suggested allelopathic effects of *L. camara*. Our study was conducted in a semi‐natural environment by growing crops in pots filled with soil samples from agriculture fields. These variable experimental results, even though allelopathic compounds were rarely measured in these studies, suggest that allelopathic effects of *L. camara* on crop plants are context dependent.

### 4.2 Legacy effects

We tested whether *L. camara* changes soil properties, such as soil nutrients and microorganisms, of soil samples with different histories of *L. camara* invasion and consequently may have lasting effects on crop growth. Farmers in the study area claim that *L. camara* affects soil nutrients by enriching the soil (A.H., pers. obs.). In our study the C:N ratio and organic matter content were higher in non‐invaded soils than in soils where *L. camara* was growing, while total N, P, K and other chemical parameters did not differ. The high organic matter content, and related higher C:N content in non‐invaded soils in our study could be a result of the use of organic manure in these fields, that were actively cultivated, and may not have been affected by presence of *L. camara*. This would be similar to the results of a study in Australian sub‐tropic rainforest and open Eucalyptus stands revealed no differences in soil N, P and K between *L. camara* invaded and non‐invaded soils (Osunkoya & Perrett, 2011). However, it is also possible that the higher C.N ratio and the higher organic matter content in non‐invaded locations indicates that *L. camara* promotes nutrient cycling, decomposition of soil organic matter, and increased respiration (Fan et al., 2010). Indeed, some other studies have revealed differences in soil nutrient content between invaded and non‐invaded sites which were associated to *L. camara* invasion. Higher pH, Mg, Ca and K were found in *L. camara* invaded areas of forest, shrub‐grassland and riverine habitat compared to uninvaded areas in Nairobi national park (Simba et al., 2013). Higher N content was also found in *L. camara* invaded shrubland and grassland in a dry deciduous forest compared to non‐invaded areas in India (Sharma & Raghubanshi, 2009). However, the lower belowground fresh crop biomass in uninvaded soil may be the result of higher N availability, which we did not measure. It is possible that total N is not a good proxy to demonstrate increased nutrient turnover, while changes in OM and C:N ratio are. Moreover, the effect of plant invasion on soil is often site and species specific, which may explain the different findings (Dassonville et al., 2008; Ehrenfeld, 2003; Pyšek et al., 2012; Stefanowicz et al., 2016) and further studies are needed to confirm the cause of the differences observed in our study.

If allelochemicals bind to soil particles, and thus persist in the soil for an extended period, they may affect plants growing in the soil after the alien plant has been removed (Del Fabbro & Prati, 2015). However, allelochemicals may decompose and be utilized by soil microbes and consequently the allelopathic effects may disappear over time (Zeng, 2014). In our study the addition of activated carbon to the soil that was previously invaded by *L. camara* had no significant effect on crop biomass. The results therefore indicate an absence of an allelopathic legacy of *L. camara*. These findings are similar to those of a study using soil from areas previously invaded and non‐invaded by 11 different invasive species in European countries, where activated carbon was added to suppress allelochemicals (Del Fabbro & Prati, 2015).

Soil microorganisms can have many effects on plant growth. For example, presence of plant growth promoting bacteria and mycorrhizal fungi may improve soil properties and organic matter content (Hayat et al., 2010; Nadeem et al., 2014), but the soil microbial community may also contain organisms that are detrimental to the growth of certain plant species (Ehrenfeld, 2003). Changes in the microbial community or the abundance of soil microorganisms are therefore likely to affect the growth of crops. This was illustrated in our second study, where a 30% increase in cassava biomass and a 90% decrease of maize biomass were observed when comparing autoclaved soils with soil with 5% non‐autoclaved soil, which added microorganisms from the eighteen fields where the soil was collected. Zhang et al., (2011) found in a pot experiment that maize grew significantly larger on sterilized than on unsterilized soil. Interestingly, they found that mycorrhizal colonization of maize was higher when grown on sterilized soil. Cassava is highly P limited on sterilized soil and presence of mycorrhizae increases P uptake and growth (Howeler, 2002). The results of these studies suggest that sterilization reduces negative effects of soil microorganisms on maize growth and reduces AM colonization in cassava, which may explain the results obtained in our study. Yet, the effects of microorganisms on crop growth was irrespective of the soil types with different *L. camara* invasion histories, indicating that *L. camara* does not affect growth of maize and cassava through changes in the soil microbial community. The opposing responses of maize and cassava to the presence of microorganisms suggest different relationships between these crops and the soil microbial community. One reason for these differences could be the presence of plant pathogens that affect maize, but not cassava or less so. Another reason may have been a higher cost associated with the formation of mycorrhizas for maize than cassava. Cassava depends on mycorrhizal fungi, but larger cuttings grow faster than small cuttings, apparently as a result of the larger reserves (Habte & Byappanahalli, 1994). As we used large cuttings when planting cassava and seeds for planting maize in our study, it is possible that the maize plants had to invest earlier in mycorrhizas than the cassava plants, which then reduced their growth rate.

## 5 CONCLUSION

Most previous studies of the impacts of *L. camara* were done under conditions that do not reflect realistic interactions between *L. camara* and native plants, such as addition of aqueous extracts or ground *Lantana* tissue to Petri dishes or soil. Our study is unique, because we assessed the effect of the presence of live *L. camara* on crops growing in the same soils. Because of the methodological difference it is not surprising that our results do not confirm the results of previous studies that indicated allelopathic effects of *L. camara*. However, our results demonstrate the need for realistic experimental conditions when assessing impacts of IAS on crop growth: although we were unable to identify the mechanism underlying the observed impact, our experimental approach allowed us to identify when impacts occur under semi‐natural conditions, which indicates when management could be undertaken to minimize these effects.

Our two studies aimed to disentangle immediate and legacy effects, as well as direct and indirect effects of *L. camara* on the growth of two major staple crops in East Africa, which may have implications for the management of *L. camara* in agricultural fields. Farmers state that increases soil nutrient content, resulting in a fertilizer effect once the plants are removed, but also that *L. camara* reduces crop growth. To our knowledge there is no study that has shown the beneficial effect of *L. camara* alleged by the farmers in East Africa (Shackleton et al., 2017). Our study also did not reveal beneficial effects of *L. camara* on soil nutrients or crop growth, but the results revealed that *L. camara* can have significant direct negative impacts on the growth of some crops: while maize biomass was significantly reduced in the presence of *L. camara*, the biomass of cassava was not affected. As there were no legacy effects, the results suggest that removal of *L. camara* in croplands is sufficient to remediate their negative effects. Future studies where *L. camara* is experimentally removed from crop fields are needed to confirm this.

## CONFLICT OF INTEREST

The authors declare they have no competing interests.

## Supporting information


Appendix S1

## Data Availability

Upon acceptance of the manuscript for publication, all data will be made available through Dryad or ckan.cabi.org.
