# Supplementary material for: Impact of invasive Lantana camara on maize and cassava growth in East Usambara, Tanzania
Source: Plant Environ Interact. 2022 Sep 24;3(5):193–202. doi: 10.1002/pei3.10090 (PMC10168096; doi:10.1002/pei3.10090)

Supporting Information to Hamad et al. “Impact of invasive Lantana camara on maize and cassava growth in East Usambara, Tanzania”.

SI1. Correlation matrix, depicting the strength and direction of correlations (correlation efficients) among soil variables. Strength is indicated by the size of the circles; positive coefficients are indicated by blue and negative coefficients by red colouring of the circles.


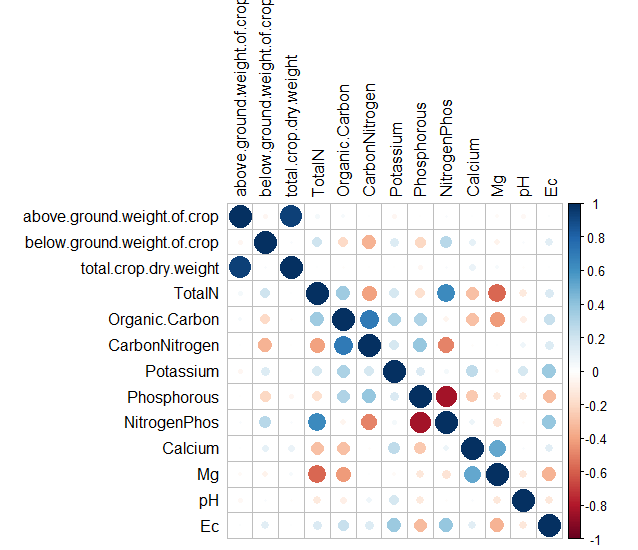

Supplement: Supplementary file 1 — Appendix S1 [file PEI3-3-193-s001.docx]
